# Perception of oral health and medical conditions as possible predictors of oral health status in visually impaired adolescents: a cross‐sectional study

**DOI:** 10.1186/s12903-021-01447-w

**Published:** 2021-02-27

**Authors:** Nasrin Sharififard, Katayoun Sargeran, Mahdia Gholami

**Affiliations:** 1 Department of Community Oral Health, Research Center for Caries Prevention, Dentistry Research Institute, School of Dentistry, Tehran University of Medical Sciences, Tehran, Iran; 2 Department of Community Oral Health, School of Dentistry, Tehran University of Medical Sciences, North Kargar Ave, 1439955991 Tehran, Iran

**Keywords:** Oral health, Visually impaired, Adolescents, School children, DMFT, Iran

## Abstract

**Background:**

We aimed to assess oral health and oral hygiene status among visually impaired adolescents and also to evaluate the factors related to their oral health.

**Methods:**

This was a cross-sectional study among 130 visually impaired adolescents in Tehran, Iran, in December 2018. Of three schools for visually impaired children in Tehran, children in the sixth to the tenth grade (aged 12–17 years) were included after obtaining their parents’ consent. WHO oral health questionnaire for children was filled out through face to face interviews. General characteristics were age, gender, status of visual impairment, place of residence, and parental education level. Oral health-related questions were perceived dental and gingival health, dental self-care, dental visits, medical conditions, and dietary habits. Oral examinations included Decayed, Missing, and Filled Teeth (DMFT) index, the Simplified Oral Hygiene Index (OHI‑S), and Bleeding on Probing (BOP). Univariate and multiple logistic regression tests were applied using STATA.

**Results:**

The mean age (SD) of the adolescents was 14.45 (1.61) years. The fully blind children were 33.8%, and those with low vision were 66.2%. Regarding the perception of dental and gingival health, nearly half of the adolescents were satisfied. The mean DMFT and decayed component (DT) were 2.43 ± 2.24 and 1.92 ± 2.12, respectively. The frequency of DMFT ≥ 3 was 45.4%. The mean OHI-S was 2.01 ± 0.70, and OHI-S > 1.8 was reported in 60% of children. The prevalence of BOP was 79.2%. DMFT was significantly associated with toothache (*P* = 0.003, OR = 3.70, 95% CI: 1.54–9.09), perceived dental health (*P* = 0.005, OR = 3.06, 95% CI: 1.40–6.67), and medical conditions (*P* = 0.03, OR = 3.13, 95% CI: 1.13–8.68). In addition, OHI-S was related to gender (*P* = 0.02, OR = 0.36, 95% CI: 0.15–0.83), perceived dental health (*P* = 0.006, OR = 2.87, 95% CI: 1.35–6.12) and medical conditions (*P* = 0.04, OR = 3.05, 95% CI: 1.04–8.97). BOP was associated with perceived gingival health (*P* = 0.02, OR = 2.94, 95% CI: 1.18–7.33).

**Conclusions:**

Medical conditions and perceived dental and gingival health are possible predictors for oral health status in these adolescents. Although these children could not visualize caries or gingival bleeding during the brushing time, they could perceive the status of their oral health correctly. Also, being involved in medical problems can make more ignorance of oral health.

## Background

Visual impairment is an increasing disability, especially in developing countries. In 2020, at least one billion people suffer from vision impairment which could have been prevented or has not received enough attention [1]. Population growth and aging will increase the risk of vision impairment [1]. The prevalence of distance vision impairment in low- and middle-income regions was about four times higher than in high-income regions in 2015 [2]. In Iran age-standardized prevalence of vision loss was estimated at 12.3% in 2015 [3].

Visually impaired children face some difficulties in oral health maintenance. They cannot visualize visible dental caries or whether gingival bleeding exists during tooth brushing, which consequently affects their oral health status and early dental visit [4]. In previous studies, poor oral hygiene [5–7], and gingival bleeding [5, 8–11] were frequently observed in visually impaired children. High prevalence of dental caries [5], and frequent trauma to the anterior teeth were also reported for these children [7, 8, 12]. Different factors, such as lack of oral health knowledge [5, 13], improper oral hygiene behavior [5], and irregular dental visits [5, 7, 9] may cause these problems.

In comparison with the general population, the visually impaired children had poorer oral health status [5], and poorer oral hygiene [5, 12, 14–16].

In Iran, studies for visually impaired children are very limited. A study about the oral health of 4–12-year-old visually impaired children showed the mean total DMFT (DMFT + dmft) was 3.7 ± 3.2, and the mean dmft was 4.1 ± 4.3. Normal gingival status without inflammation was observed only in 14.4% of children [17]. Another study in school children showed that 55% of participants had poor oral hygiene, and among them, 22.7% had dental caries [18].

A study showed visual impairment had psychological implications, including sadness, anxiety, and depression [19]. In addition, visually impaired children had more medical conditions compared with sighted peers [5]. Thus it is necessary to assess the impact of medical conditions (other diseases) on the oral health of visually impaired children.

Oral Health-Related Quality of life (OHRQoL) is affected by several factors in adolescents. These include carious lesions left untreated, specifically lesions with toothache. Moreover, the children’s physical and psychological development as well as school and daily-life achievements may be affected heading to lower OHRQoL [20].

Adolescence is the age of experiencing autonomy. Therefore, it is a critical period in which adolescents socialize more and act more independently with respect to their health-related issues [21] With a common increase in BMI during adolescence [22], the consumption of junk food and drinks high in sugar increases during this period [23], especially soft drinks [24, 25]. The pattern of beverage intake in Iranian adolescents is not favorable [26]. Oral health habits such as brushing and dental visits decrease from childhood to adolescence [27]. Thus suitable oral hygiene, healthy dietary patterns, and regular dental check-ups should be promoted during this period [20].

According to the available data, no study was conducted about the oral health of visually impaired adolescents in Iran. Moreover, the relation between oral health-related factors such as self-perceived oral health, dietary habits, and medical conditions with oral health status is still not clarified for this group. Therefore, in the current study, we aimed to assess oral health (DMFT and BOP), and oral hygiene status (OHI-S), among 12–17-year-old, visually impaired school children and evaluate the factors which related to them.

## Methods

### Design, setting, and eligibility criteria

The present study was an analytical cross-sectional study among 130 visually impaired adolescents in Tehran, Iran, conducted in December 2018. Pilot and data collection was done from December 2018 to January 2019. Inclusion criteria were being in the sixth to the tenth grade (aged 12–17 years) and having the parents’ consent. Children were excluded from the study if they were not cooperative, and/or had mental disabilities or were absent at the time of the study. In Iran, girls and boys study in separate schools. There are only three schools for visually impaired children in Tehran which visually impaired children of all districts or even other cities study in. One of these three schools was for girls located in the north of Tehran with 98 students (school 1). The two others were for boys (school 2 and 3): school 2 located in the center of Tehran with 53 students and school 3 located in the west of Tehran with 150 students. Of all three schools for visually impaired children in Tehran, total of 133 children aged 12–17 years were recruited (census), which all had parents’ consent and only three children (2%) were excluded (one student from each school) because they were absent during the research period. All the participants were cooperative, none of them had mental disabilities and none of them refuse to join the study. Thus the inclusion rate of the adolescents of these three schools was 98%. Ultimately 130 adolescents participated in this study. The participants in each school were as follow: school 1, for girls (n = 37); school 2, for boys (n = 25); school 3, for boys (n = 68).

### Study variables and data collection

World Health Organization (WHO) oral health questionnaire for children [28], including oral health-related questions and general characteristics, was filled out through face to face interviews. For every participant, the questions were read one by one followed by the optional answers. If it was necessary, the interviewer repeated the items to understand well. For content validity of the questionnaire for visually impaired children, the questionnaire was filled out for 20 visually impaired children in the eleventh grade and we asked them if there is a need for any changes in the questions according to their special conditions. Finally, two oral health experts reviewed and confirmed the questionnaire validity. The questionnaire showed an acceptable validity with Cronbach alpha coefficient of more than 0.9.

Oral examinations were implemented to assess Decayed, Missing, and Filled Teeth (DMFT), the Simplified Oral Hygiene Index (OHI‑S), and Bleeding on Probing (BOP). The school children were examined with a headlamp, a disposable mouth mirror and explorer, a WHO periodontal probe, and disposable gloves and masks while they were sitting on a chair in a comfortable position.

#### General characteristics

The children’s age, gender, grade, the status of visual impairment, place of residence, and their fathers’ and mothers’ education were considered as the general characteristics.

According to the 11th revision of International Classification of Diseases (ICD-11), the severity of vision impairment is classified into six levels: mild vision impairment, moderate vision impairment, severe vision impairment, and three categories for blindness [29]. In this study, we categorized visual impairment status in two groups: “low vision” for moderate and severe vision impairment, and “blind” for blindness [8].

Fathers’ and mothers’ education data were collected according to the number of years they spent in school and university. Since after 12 years of education in school, people would get a high-school diploma in Iran, in the analysis, we categorized them into two groups: (1) <12 years (less than a high-school diploma); (2) ≥12 years (a high-school diploma or higher).

#### Oral health-related questions

Oral health-related questions included the following variables:

- perceived dental health status (how would you describe the health of your teeth?),
- perceived gingival health status (how would you describe the health of your gums?),

In both “a” and “b” the 7-point Likert scale responses were categorized into two groups: (1) satisfied (excellent, very good, and good); (2) not satisfied (average, poor, very poor, and don’t know) for the analysis.

(iii) the frequency of tooth brushing (How often do you clean your teeth?) was dichotomized into (1) at least once a day; (2) less than once a day,
(iv) using a toothbrush, toothpaste, and dental floss,
(v) using fluoridated toothpaste (Do you use toothpaste that contains fluoride?) with three responses (1. yes; 2. no; 3. don’t know),
(vi) dental visit frequency in the last 12 months (How often did you go to the dentist during the past 12 months?), which were coded as: (1) yes (for at least once); (2) no (not in the past 12 months),
(vii) the reason for the dental visit in the last 12 months,
(viii) medical conditions dichotomized into two groups: (1) healthy (if there were no other physical or psychological diseases along with visual impairment); (2) not healthy (if there were one or more other physical or psychological diseases along with visual impairment), and (i) dietary habits included 9 questions concerning the consumption of sugary snacks or drinks (fruits, biscuits, cakes, cream, soft drinks, jam and honey, gum with sugar, sweets and candy, milk with sugar, tea with sugar, and coffee with sugar).

In order to evaluate the association between frequency of sugar consumption and oral health status, the answers were scored as follows: several times a day = 6, every day = 5, several times a week = 4, once a week = 3, several times a month = 2, never = 1. Then the sum of the scores for these nine questions was calculated for each child. After that the scores changed into scale 0–100 [30]. Normality test of the scores showed normal distribution (Shapiro–Wilk test: *P* = 0.13). Because of normal distribution of sugar consumption score, mean and median values were very close to each other (mean = 33.69, median = 33.33). So, choosing each one as the cut-off point had the same result [31]. In this study, we considered the median score of sugar consumption as the cut-off point between the low and high sugar consumption [32]. Consequently, according to the median score of sugar consumption among 130 study participants, children were categorized into two groups: *1*. low sugar consumption, *2*. high sugar consumption. Participants scoring more than the median score were identified as having high sugar consumption while participants scoring less than or equal to the median score were considered to have low sugar consumption.

#### The calibration

Oral examinations were carried out by a single examiner. For intra-examiner calibration, 10 children from the 11th grade were examined. Between the two assessments of BOP, there was half an hour break time to resolve previous bleeding (Kappa = 1). The examiner also assessed debris index (DI) and calculus index (CI) for OHI-S. Due to the removal of the debris by the explorer during the first examination, it was not possible to assess DI again. Thus only for CI, intra-class correlation coefficient (ICC) was calculated (ICC = 1). The examiner was calibrated by the WHO Oral Health Surveys Basic Methods for DMFT assessment (33). ICC for DMFT was 0.90.

#### Clinical examinations

Caries experiences assessed through DMFT index were dichotomized into two groups (DMFT < 3, and DMFT ≥ 3) [20, 34]. Oral hygiene was assessed by OHI-S [35], which consisted of DI and CI. Six permanent tooth (3, 8, 14, 19, 24, and 30) surfaces were scored on a scale of 0 to 3. The mean scores indicated as DI and CI [35, 36]. The sum of DI and CI was defined as OHI-S, which was then categorized into two groups for analysis: (OHI-S ≤ 1.8, and OHI-S > 1.8). Since, in the present study, only 6.9% of the children had poor OHI-S (> 3), and none of them had poor CI (> 1.8), we dichotomized OHI-S based on the scoring for poor DI (> 1.8) [8].

To assess BOP, buccal and mediobuccal surfaces of all teeth in the first to the fourth quadrants were examined by the WHO periodontal probe. The presence of local or general bleeding was coded as yes, and the absence of bleeding was indicated as no [37].

### Statistical analyses

The STATA software version 14 (Stata Corporation, College Station, TX, USA) was used for data analysis. Univariate regression test was applied to identify the association between independent variables and categorical outcomes. To consider cluster effect of schools, we used mixed effect model in which clusters entered as random effect in the model. Multiple logistic regression analysis was performed to identify which variables were associated with outcomes in the presence of other variables to indicate possible predictors. If the *P* value for the association of variables was less than 0.2 by testing with univariate, it was included in the multiple regression analysis [38, 39]. A *P* value < 0.05 was considered statistically significant.

### Ethics statement

The Tehran University of Medical Sciences Ethics Committee approved the study (IR.TUMS.DENTISTRY.REC.1397.104). Before the study written informed consent was obtained from the parents. In addition, the children gave “assent” to be sure they were happy to participate in the study.

## Results

### Descriptive results

#### General characteristic

In the three schools for blinds in Tehran, all 133 adolescents (12–17 years old) agreed to participate in the study. However, three children were excluded because they were absent during the data collection time. Ultimately, the data for 130 children were analyzed.

The mean age (SD) of the children was 14.45 (1.61) years (range: 12–17 years). The study participants included 93 male (71.5%) and 37 female children (28.5%). Fully blind children comprised 33.8% of the respondents, and the rest of the children had a low vision (66.2%). More children (68.5%) resided at home than the dormitory. About half of the participants’ mother or father were educated less than a high-school diploma (Table 1).

**Table 1:** General characteristics of 12–17-year-old visually impaired school children (n = 130)

Variable	N	%
*Gender*		
Male	93	71.5
Female	37	28.5
*Place of residence*		
Dormitory	41	31.5
Home	89	68.5
*Status of visual impairment*		
Blind	44	33.8
Low vision	86	66.2
*Father’s education*		
<12 years^a^	65	50
≥12 years^b^	54	41.5
Missing	11	8.65
*Mother’s education*		
<12 years^a^	66	50.8
≥12 years^b^	56	43.1
Missing	8	6.2

^a^Less than a high-school diploma

^b^A high-school diploma or higher

#### Oral health‐related variables

About half of the children were satisfied with their dental (49.2%) and gingival health (50%). Among the children, 47.7% brushed their teeth once a day. Only 15.4% brushed their teeth twice and more. Some children (7.7%) reported that they never brush their teeth. Most of the children used toothbrushes and toothpaste (94.6%, and 90%, respectively). However, only 20.8% reported using dental floss to clean the interdental spaces. Among all the participants, 65.4% had no knowledge if their toothpaste contained fluoride. Some children (30%) often or occasionally experienced toothache in the last year. Among children, 13.8% never had a dental visit in their life. Only 30.8% had a dental visit in the last 12 months, which more than half of them reported pain was the main reason for their dental visit.

Most of the children (N = 106, 81.5%) were in good medical conditions (healthy). However, 18.5% of the participants had medical problems including auditory impairment (n = 3, 2.31%); heart problem, epilepsy, gastrointestinal disease, kidney disease (for each disease n = 2, 1.54%); anemia, diabetes, favism, migraine, cancer, Wolfram syndrome, hypothyroidism, asthma, Wegener’s syndrome, hydrocephaly (for each disease n = 1, 0.77%); and other diseases (n = 3, 2.31%). Children reported daily consumption of fruit (n = 80, 61.5%), biscuits, cakes and cream (n = 37, 28.5%), jam or honey (n = 30, 23.1%), tea with sugar (n = 66, 50.8%), and other nutritional items (≤ 4%). The range of sugar consumption score was 2.22–68.89. Categorizing to low and high sugar consumption according to the median score among participants (median = 33.33), showed that 56.2% (n = 72) had high sugar consumption.

### Clinical examinations

#### Caries experience

The average scores of DMFT and DT (SD) in this study were 2.43 ± 2.24 and 1.92 ± 2.12, respectively. The prevalence of caries experience was 72.3% (n = 94). DMFT for approximately half of the participants (45.4%) was three or more (DMFT ≥ 3). The DT formed the largest contribution to DMFT. Only 27.7% of the children were caries-free, and 62.3% had at least one decayed tooth. Also, 3.1% had at least one missed tooth, and 20.8% had at least one filled tooth (Table 2).

#### Oral hygiene

The mean OHI-S (SD) was 2.01 (0.70). Of all the participants, 41.5% had fair DI (0.7–1.8). Also, 92.3% had good (0-0.6), and none of them had poor CI (1.9–3). OHI-S > 1.8 was reported in 60% (n = 78) of the children (Table 2).

#### Bleeding on probing

The prevalence of BOP was 79.2% among the children (Table 2).

**Table 2:** Clinical characteristics of 12–17-year-old visually impaired school children (n = 130)

Variable	N	%
*BOP*		
Yes	103	79.2
No	27	20.8
*DMFT*		
<3	71	54.6
≥ 3	59	45.4
Caries free	36	27.7
*OHI-S*		
≤1.8	52	40
>1.8	78	60
Good (0–1.2)	18	13.8
Good or fair (0–3)	121	93.1
*DI*		
Good	0	0
Good or fair (0–1.8)	54	41.5
DI = 0	0	0
*CI*		
Good (0–0.6)	120	92.3
Good or fair (0–1.8)	130	100
CI = 0	97	74.6

	Mean (SD)	Range
OHI-S	2.01 (0.70)	0.83–4.33
DMFT	2.43 (2.24)	0–10
DT	1.92 (2.12)	0–10
MT	0.05 (0.30)	0–3
FT	0.46 (1.05)	0–4

*BOP* bleeding on probing, *OHI-S* oral hygiene index-simplified, *DMFT* decayed, missing and filled teeth, *DT* decayed teeth, *MT* missing teeth, *FT* filled teeth, *DI* Debris index, *CI* Calculus index

### Analytical results

In order to identify factors related to oral health status, we analyzed the data collected using the questionnaire with univariate test. The results showed that gender (*P* = 0.04, OR = 2.22, 95% CI: 1.02–4.83) (Table 3), toothache in the past 12 months (*P* = 0.001, OR = 4.15, 95% CI: 1.85–9.28) and perceived dental health (*P* = 0.001, OR = 3.64, 95% CI: 1.76–7.55) (Table 4) significantly affected the status of dental caries in visually impaired schoolchildren. The frequency of DMFT ≥ 3 was higher in girls than in boys (Table 3). Also OHI-S > 1.8 was associated with gender (*P* = 0.04, OR = 0.45, 95% CI: 0.20–0.97) (Table 3), and perceived dental health (*P* = 0.009, OR = 2.63, 95% CI: 1.28–5.43) (Table 4). OHI-S > 1.8 was less frequent in girls than in boys (Table 3). The prevalence of BOP was higher among children whose perceived gingival health status was reported as “not satisfied” (*P* = 0.02, OR = 2.94, 95% CI: 1.18–7.33) (Table 4). Partially blind children showed higher DMFT than fully blind but the difference was not significant (*P* = 0.07) (Table 3).

**Table 3:** Association of DMFT/dmft, OHI-S, BOP with demographic variables in 12–17 visually impaired school children (n = 130)

Variable	N (%)	DMFT ≥ 3			OHI-S > 1.8			BOP (yes)		
		*P* value	OR	95% CI	*P* value	OR	95% CI	*P* value	OR	95% CI
*Gender*										
Male^a^	93 (71.5)	0.04^b^	2.22	1.02–4.83	0.04^b^	0.45	0.20–0.97	0.53	0.75	0.30–1.86
Female	37 (28.5)									
*Place of residence *										
Dormitory^a^	41 (31.5)	0.54	1.26	0.60–2.66	0.89	0.94	0.44–2.01	0.49	1.37	0.56–3.32
Home	89 (68.5)									
*Status of visual impairment*										
Blind	44 (33.8)	0.07	0.49	0.23–1.05	0.54	1.26	0.60–2.66	0.60	1.28	0.51–3.20
Low vision ^a^	86 (66.2)									
*Father’s education*										
< 12	65 (50)	0.70	1.15	0.56–2.39	0.80	0.91	0.44–1.89	0.23	1.72	0.71–4.18
≥ 12^a^	54 (41.5)									
Missing	11 (8.5)									
*Mother’s education*										
< 12	66 (50.8)	0.18	1.64	0.80–3.38	0.83	0.92	0.45–1.90	0.79	1.12	0.48–2.64
≥ 12^a^	56 (43.1)									
Missing	8 (6.2)									

^a^Reference category

^b^Statistically significant

**Table 4:** Association of DMFT/dmft, OHI-S, BOP self-assessment and behavioral factors in 12–17 visually impaired school children (n = 130)

Variable	N (%)	DMFT ≥ 3			OHI-S > 1.8			BOP (yes)		
		*P* value	OR	95% CI	*P* value	OR	95% CI	*P* value	OR	95% CI
*Brushing frequency*										
At least once a day ^a^	82 (63.1)	0.66	1.18	0.57–2.40	0.24	1.56	0.74–3.29	0.19	1.89	0.73–4.87
Less than once a day	48 (36.9)									
*Perceived dental health*										
Satisfied ^a^	64 (49.2)	0.001^b^	3.64	1.76–7.55	0.009^b^	2.63	1.28–5.43	0.11	2.03	0.85–4.84
Not satisfied	66 (50.8)									
*Perceived gingival health*										
Satisfied^a^	65 (50)	0.22	1.55	0.77–3.10	0.07	1.91	0.94–3.89	0.02^b^	2.94	1.18–7.33
Not satisfied	65 (50)									
*Toothache in the past 12 months*										
Often, occasionally	39 (30)	0.001^b^	4.15	1.85–9.28	0.31	1.50	0.68–3.29	0.60	1.29	0.49–3.35
Rarely, never, don’t know^a^	91 (70)									
*Dental visit in the past 12 months*										
Yes^a^	40 (30.8)	0.75	0.88	0.42–1.87	1	1	0.47–2.14	0.75	1.16	0.47–2.87
No	90 (69.2)									
*Reason for the last dental visit*										
Pain or trouble	22 (55)	0.73	1.25	0.36–4.36	0.90	0.92	0.26–3.28	0.47	1.73	0.39–7.72
No pain^a^	18 (45)									
*Sugar consumption*										
Low^a^	57 (43.8)	0.96	0.98	0.49–1.97	0.66	1.17	0.58–2.37	0.94	1.03	0.44–2.42
High	73 (56.2)									
*Medical conditions*										
Healthy^a^	106 (81.5)	0.07	2.35	0.94–5.85	0.10	2.30	0.85–6.26	0.58	1.39	0.43–4.46
Not healthy	24 (18.5)									

^a^Reference category

^b^Statistically significant

### Multiple regression analysis of outcomes

A stepwise multiple logistic regression analysis was executed to estimate the relationship between DMFT ≥ 3, OHIS > 1.8, and BOP as dependent variables and various independent variables (Table 5). DMFT ≥ 3 showed an association with toothache in the past 12 months (*P* = 0.003, OR = 3.70, 95% CI: 1.54–9.09), perceived dental health (*P* = 0.005, OR = 3.06, 95% CI: 1.40–6.67), and medical conditions (*P* = 0.03, OR = 3.13, 95% CI: 1.13–8.68). OHIS > 1.8 had an association with gender (*P* = 0.02, OR = 0.36, 95% CI: 0.15–0.83), perceived dental health (*P* = 0.006, OR = 2.87, 95% CI: 1.35–6.12), and medical conditions (*P* = 0.04, OR = 3.05, 95% CI: 1.04–8.97). BOP had only a relation with perceived gingival health (*P* = 0.02, OR = 2.94, 95% CI: 1.18–7.33). Among these factors, having a toothache, not satisfied perceived dental and gingival health, and having medical problems, were possible risk factors while being female was a possible protective factor against OHI-S > 1.8. Possible predictors that remained in the model were illustrated in Table 5.

There was no statistically significant association between the medical conditions and DMFT (*P* = 0.07) and OHI-S (*P* = 0.10) in univariate analysis (Table 4). However, multiple logistic regression revealed that DMFT ≥ 3 and OHI-S ≥ 1.8 were statistically more frequent in children who suffered a medical problem as compared with healthy children.

**Table 5:** Multiple logistic regression analysis of factors influencing DMFT, OHI-S, and BOP (n = 130)

	Variables	OR	SE (OR)	z	*P* value	95% CI for OR
*DMFT ≥ 3*						
Step 1	Gender	2.22	0.21	− 1.72	0.08	0.90–5.55
	Status of visual impairment	0.52	0.86	1.47	0.14	0.21–1.25
	Medical conditions	2.55	1.40	1.70	0.09	0.87–7.49
	Perceived dental health	3.17	1.34	2.74	0.006	1.39–7.24
	Toothache in the past 12 months	3.45	0.13	− 2.67	0.008	1.39–8.33
	Mother’s education	1.28	0.33	− 0.59	0.56	0.55–2.94
Step 2	Gender	1.96	0.22	− 1.54	0.12	0.83–4.76
	Status of visual impairment	0.53	0.83	1.44	0.15	0.22–1.27
	Medical conditions	2.51	1.35	1.71	0.09	0.87–7.19
	Perceived dental health	3.26	1.33	2.89	0.004	1.46–7.26
	Toothache in the past 12 months	3.57	0.13	− 2.79	0.005	1.45–8.33
Step 3	Gender	2.04	0.21	− 1.63	0.10	0.86–5
	Medical conditions	2.91	1.53	2.02	0.04	1.03–8.18
	Perceived dental health	3.20	1.29	2.88	0.004	1.45–7.08
	Toothache in the past 12 months	3.45	0.13	− 2.80	0.005	1.45–8.33
Step 4	Medical conditions	3.13	1.63	2.20	0.03	1.13–8.68
	Perceived dental health	3.06	1.22	2.81	0.005	1.40–6.67
	Toothache in the past 12 months	3.70	0.12	− 2.93	0.003	1.54–9.09
*OHI-S > 1.8*						
Step 1	Gender	0.36	1.19	2.35	0.02	0.16–0.85
	Medical conditions	3.10	1.72	2.04	0.04	1.05–9.19
	Perceived gingival health	1.27	0.54	0.56	0.58	0.55–2.94
	Perceived dental health	2.58	1.11	2.21	0.03	1.11–5.99
Step 2	Gender	0.36	1.21	2.40	0.02	0.15–0.83
	Medical conditions	3.05	1.68	2.02	0.04	1.04–8.97
	Perceived dental health	2.87	1.11	2.73	0.006	1.35–6.12
*BOP (yes)*						
Step 1	Brushing frequency	1.48	0.75	0.78	0.44	0.55–3.98
	Perceived dental health	1.26	0.63	0.46	0.64	0.47–3.39
	Perceived gingival health	2.46	1.27	1.74	0.08	0.89–6.79
Step 2	Brushing frequency	1.53	0.76	0.85	0.39	0.57–4.07
	Perceived gingival health	2.71	1.29	2.10	0.04	1.07–6.87
Step 3	Perceived gingival health	2.94	1.37	2.32	0.02	1.18–7.33

*SE* standard error

## Discussion

Caries experience, oral hygiene, and gingival bleeding in visually impaired adolescents were associated with perceived dental or gingival health, medical conditions, toothache, and gender.

In the present study, most of the children used toothbrush and toothpaste, but rarely used dental floss, which is in accordance with previous studies [14, 15, 40, 41]. However, daily brushing occurred less frequently in our study compared with most other studies [14, 15, 40]. Despite the fact that all kinds of toothpaste contain fluoride in the Iranian market, most of the children had no idea if their toothpaste contained fluoride, which showed a lack of knowledge in this regard.

Our results showed that toothache had the greatest association with DMFT, which was in line with previous studies [9, 42]. We found that most of the children had an irregular dental visit unless they had to have a dental appointment due to toothache. A cohort study showed that the percentage of children visiting the dentist at least once a year decreased with age, especially in late adolescence [43]. These results are consistent with the findings of Parkar and colleagues who showed the decay component formed the major component of the index, while the filled component was almost negligible [15]. We speculated this could be associated with difficulty in access to dental facilities and the cost of treatments, which indicates the necessity of special prevention programs for these children.

A high proportion of decayed teeth observed in the present study is in line with previous studies conducted for visually impaired children [7, 8, 14, 40, 42]. The prevalence of caries-free in our study (27.7%) is comparable to caries-free children (7–16-year-old) in Turkey (26.40%) [6].

Despite the fact that 93.1% of the visually impaired adolescents had good or fair oral hygiene status, a high prevalence of gingival bleeding was observed in this study, which is in line with previous studies [9, 11, 40]. In a national oral health survey conducted on the Iranian population in 2011–2012, gingival bleeding was observed among 26.9% of 12-year-old children and 33.8% of 15-year-old children [44]. Accordingly, gingival bleeding in visually impaired children (79.2%) is more frequent than the general Iranian population. We speculated that the high rates of gingival bleeding in visually impaired children could be associated with inappropriate tooth brushing in all sites, which could result in the insufficient clearing of teeth.

In the present study, DMFT was higher in girls in univariate analysis, which is in line with a study in China [9] but in contrast with the study by Solanki and co-workers. Also, according to the last national survey in Iran (2012), girls had higher DMFT than boys in both 12- and 15-year-old general population [44].

Better oral hygiene in girls in our study is consistent with the report by Parkar and colleagues [15], which can be in agreement with decreasing the percentage of twice tooth brushing per day with age for males and increasing with age for females in sighted children [43].

In our study, 18.5% of children had medical problems. This finding is comparable to a study by Alsadhan and others [5]. Medical conditions showed significant association with caries experience and oral hygiene. This association indicated that when the main focus is kept predominantly on managing medical problems, oral health is mostly neglected. Thus, oral health care should be approached jointly with general health care in order to achieve a more holistic view of these conditions.

There are too limited data about dietary habits and their relations with oral health in visually impaired children. Suresan and colleagues found an association between DMFT/dmft and solid or sticky sugars [7]. However, no association between DMFT and the frequency of sugar consumption was found in our study, which was in agreement with results reported by Wening and co-workers for sighted children [45] due to multifactorial features of caries. A study on sighted adolescents in Iran showed sweetened soft beverages had been consumed by 25.6% of the participants with a significant effect on DMFT [26]. However, in our study, only one child reported daily use of soft drinks. Maybe it is because visually impaired children had less chance to demand snacks or soft drinks in the market, so restricting sugar consumption for them is easier than the general population. Tehran is a multicultural city which people emigrate from different areas of the country to live there. In addition, some of the students who resided at dormitory, were from other cities. So we expect the study results could be generalized to all schools for visually impaired children in Iran. However, conducting further studies at national level is recommended to check for wider generalizability.

### Limitations

The use of a cross-sectional design to investigate associations, the small sample size and the lack of comparison group were some of the limitations of this study. In addition, through the face to face interview, the participant may hide the truth to present him/herself in a good manner. The use of the median of the sugar consumption to categorize low and high sugar consumption is another limitation as this cut-off would be only applicable to the studied population, which limits comparison with other studies. Meaningful comparisons between previous studies are limited because most of them have been conducted in a wide age range (usually all grades in schools) and in different countries with different cross-cultural differences in living standards, dietary habits, and genetic predisposition. Consequently, more studies for visually impaired adolescents are needed to further verify our findings.

#### Strengths

Most of the studies for visually impaired school children are conducted in a wide age range or in a small population. The present study was designed for visually impaired adolescents with an appropriate sample size to assess their oral health status.

## Conclusions

Medical conditions and perceived dental and gingival health are possible predictors for oral health status in visually impaired adolescents. Although they could not visualize caries or gingival bleeding during brush time, they could perceive the status of their oral health correctly. Also involving in medical problems can make more ignorance of oral health in this group of children.

The frequency of untreated caries indicated that it was imperative as a public health issue to be targeted by effective oral health promotion programs to improve access to oral healthcare services among these children and to increase the use of appropriate caries-preventing methods along with medical care.

## Data Availability

The datasets used and analyzed during the current study are available from the corresponding author on reasonable request.

## Abbreviations

OHI‑S: Oral Hygiene Index‑Simplified
DMFT: Decayed, missing, and filled teeth
BOP: Bleeding on probing
CI: Calculus index
DI: Debris index
DT: Decayed teeth
MT: Missing teeth
FT: Filled teeth
STATA: Stata Corporation, College Station, TX, USA
*P*: *P* value
ICC: Intra-class correlation coefficient
95% CI: 95% confidence interval
OR: Odds ratio
SD: Standard deviation
ICD-11: The 11th revision of international classification of diseases
WHO: World Health Organization
OHRQoL: OHRQoLOral Health-Related Quality of Life
